# Intestinal permeability before and after albendazole treatment in low and high socioeconomic status schoolchildren in Makassar, Indonesia

**DOI:** 10.1038/s41598-022-07086-7

**Published:** 2022-03-01

**Authors:** Aldian I. Amaruddin, Jan Pieter R. Koopman, Munawir Muhammad, Kaatje Lenaerts, Hans M. H. van Eijk, Eric A. T. Brienen, Anoecim R. Geelen, Lisette van Lieshout, Sitti Wahyuni, Ed J. Kuijper, Romy D. Zwittink, Firdaus Hamid, Erliyani Sartono, Maria Yazdanbakhsh

**Affiliations:** 1 Department of Parasitology, Faculty of Medicine, Hasanuddin University, Makassar, 90245 Indonesia; 2 Department of Parasitology, Leiden University Medical Center, PO Box 9600, 2300 RC Leiden, The Netherlands; 3 Department of Pharmacology, Faculty of Medicine, Hasanuddin University, Makassar, Indonesia; 4 Department of Surgery, NUTRIM School of Nutrition and Translational Research in Metabolism, Maastricht University Medical Centre, 6229 HX Maastricht, The Netherlands; 5 Experimental Bacteriology, Department of Medical Microbiology, Leiden University Medical Center, 2333 ZA Leiden, The Netherlands; 6 Center for Microbiome Analyses and Therapeutics, Department of Medical Microbiology, Leiden University Medical Center, 2333 ZA Leiden, The Netherlands; 7 Department of Microbiology, Faculty of Medicine, Hasanuddin University, Jalan Perintis Kemerdekaan Km. 10 Kampus Tamalanrea, Makassar, 90245 Indonesia

**Keywords:** Microbiota, Small intestine, Epidemiology, Parasitic infection

## Abstract

Intestinal helminths are highly prevalent in low-SES children and could contribute to poor health outcomes either directly or via alteration of the gut microbiome and gut barrier function. We analysed parasitic infections and gut microbiota composition in 325 children attending high- and low-SES schools in Makassar, Indonesia before and after albendazole treatment. Lactulose/Mannitol Ratio (LMR, a marker of gut permeability); I-FABP (a surrogate marker of intestinal damage) as well as inflammatory markers (LBP) were measured. Helminth infections were highly prevalent (65.6%) in low-SES children. LMR and I-FABP levels were higher in low-SES children (geomean (95%CI): 4.03 (3.67–4.42) vs. 3.22 (2.91–3.57); *p*. adj < 0.001; and 1.57 (1.42–1.74) vs. 1.25 (1.13–1.38); p. adj = 0.02, respectively) while LBP levels were lower compared to the high-SES (19.39 (17.09–22.01) vs. 22.74 (20.07–26.12); p.adj = 0.01). Albendazole reduced helminth infections in low-SES and also decreased LMR with 11% reduction but only in helminth-uninfected children (estimated treatment effect: 0.89; p.adj = 0.01). Following treatment, I-FABP decreased in high- (0.91, p.adj < 0.001) but increased (1.12, p.adj = 0.004) in low-SES children. Albendazole did not alter the levels of LBP. Microbiota analysis showed no contribution from specific bacterial-taxa to the changes observed. Intestinal permeability and epithelial damage are higher while peripheral blood inflammatory marker is lower in children of low-SES in Indonesia. Furthermore, treatment decreased LMR in helminth-uninfected only.

## Introduction

Approximately 1.5 billion people suffer from soil-transmitted helminth (STH) infections worldwide^1^. These infections are caused by different species of worms including *Ascaris lumbricoides, Trichuris trichiura, Necator americanus* and *Ancylostoma duodenale*^2^. Children from lower socioeconomic status (SES) backgrounds are often highly infected with parasitic helminths because of poor sanitation and limited access to clean water facilities^3^. Untreated, STH infection can cause malnutrition, impaired growth and physical development^2,4^.

Elevated intestinal permeability, and therefore impaired barrier function, along with gut inflammation and dysbiosis have been observed in various pathological conditions such as in stunting, obesity, and metabolic diseases^5–8^. The human intestine, which essentially allows absorptions of dietary products while maintaining a barrier function with selective permeability, prevents intrusion of pathogens or translocation of harmful products^9^. The intestinal lining is at the interface of interaction between helminths and protozoa that reside in the gastrointestinal tract and their human host and if damaged by parasites, could lead to poor barrier function and poor health outcomes.

To quantify the intestinal permeability in vivo, assays can be used that utilise the absorptive properties of differently sized carbohydrate probes^5^. The lactulose/mannitol ratio (LMR) is the most commonly used probe combination. As a result of increased intestinal permeability, bacterial products may be able to cross the barrier more easily and end up in the systemic circulation. Therefore, another way to characterize the intestinal permeability is by looking at markers for bacterial translocation. Examples of these include lipopolysaccharide binding protein (LBP)^6^. Compromised intestinal epithelial integrity and epithelial cell damage can also be assessed by measuring markers of intestinal injury such as intestinal-fatty acid binding protein (I-FABP)^10^.

Previous studies in low to middle income countries, have shown a difference in gut permeability of children of high and low SES^11^. Although an association between helminth infections and increased intestinal permeability^12^ was found in another study, there was no confirmation of causality through treatment. In the current study, we assessed the association between socioeconomic status (SES), intestinal parasitic infections and markers of intestinal barrier function. To this end, the presence of intestinal parasitic infections and the levels of LMR, I-FABP, and LBP were determined in schoolchildren of low- and high-SES, before and after albendazole treatment. In addition, this population has been characterized for baseline gut microbiota to assess the effect of socioeconomic status^13^. Here, we assessed the alteration of the gut microbiota after albendazole treatment to delineate any contribution to intestinal permeability and barrier function.

## Results

### Study participants

A total of 325 children (165 and 160 children from low- and high-SES schools, respectively) were recruited. Fifty-four children were lost to follow-up due to migrating out of the study area, absence from school for an extended period of time, or withdrawal of consent as indicated in consort diagram in Supplementary Figure S1. There were no differences in age, sex, SES, or z-BMI between those who remained in the study and those who were lost to follow-up (Supplementary Table S1).

The characteristics of children from low- and high-SES schools are shown in Table 1. The mean age and sex were comparable in both groups. The z-BMI was higher in the high- compared to low-SES children (z-BMI, mean ± SD: 0.27 ± 1.48 vs − 0.97 ± 1.19, *p* < 0.001). Prevalence of any helminth infection in low- and high-SES was 65.6% and 1.6%, respectively (*p* < 0.001). In high-SES group, only 2 children (1.6%) were infected with *T. trichiura* and no other STH infections were detected. In low-SES children, the prevalence of *T. trichiura* and *A. lumbricoides* was 65.6% and 39.8%, respectively. No hookworm infection was detected, but 2 children (1.6%) were infected with *Hymenolepis diminuta* (Table 1).

**Table 1:** Baseline characteristics of study population for low- and high-SES schoolchildren.

Characteristics	Low-SES		High-SES		*p* value
	N	Result	N	Result	
Age in years (mean, SD)	165	10.2 ± 1.08	160	10.3 ± 0.65	0.26
**Sex (%)**					
Male	73	44.2	71	44.4	0.98
Female	92	55.8	89	55.6	
z-BMI (mean, SD)	165	− 0.97 ± 1.19	160	0.27 ± 1. 48	< 0.001
z-HA (mean, SD)	165	− 2.05 ± 1.08	160	− 0.66 ± 1.00	< 0.001
**Helminth infection (N, n, %)**					
Any intestinal helminth	128	84 (65.6)	127	2 (1.6)	< 0.001
*Ascaris lumbricoides*	128	59 (46.1)	127	0	< 0.001
*Trichuris trichiura*	128	51 (39.8)	127	2 (1.6)	< 0.001
*Hymenolepis diminuta*	128	2 (1.6)	127	0	0.16
**Intestinal protozoal infection (N, n %)**					
Any intestinal protozoa	114	83 (72.8)	120	47 (39.2)	< 0.001
*Entamoeba histolytica*	114	16 (14.0)	120	2 (1.7)	< 0.001
*Dientamoeba fragilis*	114	41 (36.0)	120	25 (20.8)	0.01
*Giardia lamblia*	114	59 (51.8)	120	28 (23.3)	< 0.001
*Cryptosporidium parvum*	114	3 (2.6)	120	0	0.07

The number of positives (n) of the total population examined (N). SD: standard deviation. Statistical testing was performed using student t-test for continuous variables and using chi-square test for categorical variables.

Similar to STH infections, intestinal protozoa prevalence was higher in low- compared to high-SES children (72.8% vs 39.2%, respectively, *p* < 0.001). The most prevalent species was *G. lamblia* followed by *D. fragilis* and *E. histolytica*. Infection with *Cryptosporidium parvum* was only detected in low-SES children (Table 1).

### Intestinal barrier function in low-SES and high-SES children at baseline

**Figure 1:**
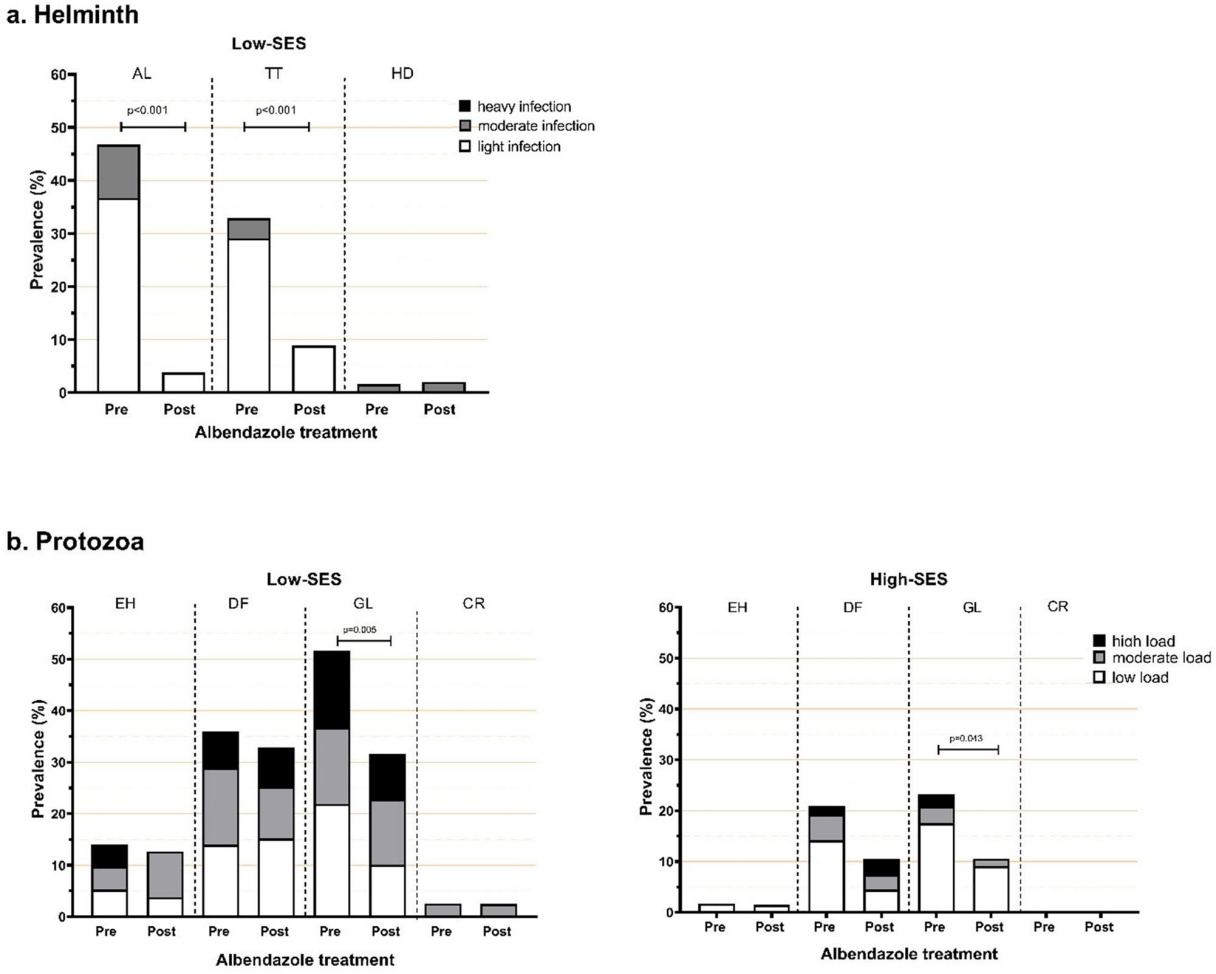
The effect of treatment on the proportion infected with (**a**) intestinal helminths (by microscopy) and (**b**) intestinal protozoa (by PCR). SES: socioeconomic status. AL: *Ascaris lumbricoides,* TT: *Trichuris trichiura,* HD: *Hymenolepis diminuta,* EH: *Entamoeba histolytica*, DF: *Dientamoeba fragilis*, GL: *Giardia lamblia*, CR: *Cryptosporidium parvum.* *p* values were calculated using a mixed effects logistic model fitted with subject random effects and adjusted for sex, age, and z-BMI.

**Figure 2:**
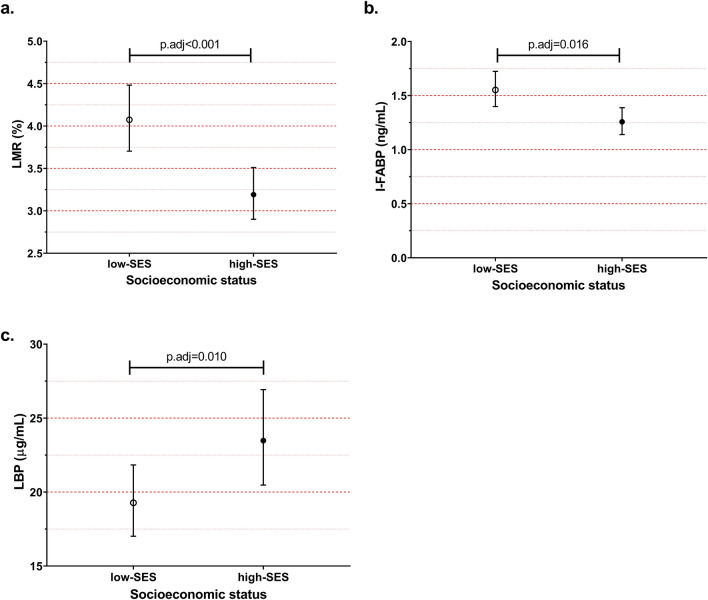
Geometric means and their 95% confidence intervals for different gut permeability markers at baseline, LMR: Lactulose Mannitol Ratio (**a**), I-FABP: Intestinal fatty acid binding protein (**b**), and LBP: Lipopolysaccharide binding protein (**c**). SES: socioeconomic status. *p* values derived from linear regression models after adjusting for age, sex, and zBMI.

Markers for intestinal permeability and acute intestinal injury exhibited substantial differences between low- and high-SES children (Figs. 1, 2). LMR was significantly higher in low- compared to high-SES children (geomean(95%CI): 4.03(3.67–4.42) vs. 3.22(2.91–3.57), respectively; adjusted *p* value (p.adj) < 0.001). I-FABP was also higher in low-SES (1.57(1.42–1.74) vs. 1.25(1.13–1.38); p.adj = 0.02). In contrast, LBP was lower in low- compared to high-SES (19.39(17.09–22.01) vs. 22.74(20.07–26.12); p.adj = 0.01). Additionally, we observed no correlation between any of these measurements (Supplementary Figure S2). To assess the role of parasitic infections in intestinal barrier function, we performed further analysis. Although univariate analysis showed that the presence of *A. lumbricoides* infection was associated with higher LMR levels (Supplementary Table S2), a subsequent linear regression model including both SES and *A. lumbricoides* demonstrated that the effect is mainly through SES (Table 2). Infection with *D. fragilis* was associated with higher LBP levels (Supplementary Table S2) but adding this variable into the model did not alter the effect of SES on LBP.

**Table 2:** Association between SES and gut permeability markers (at baseline).

Outcomes		Effect of SES on LMR, I-FABP, and LBP (GMR, 95% CI, * p* value )			
		Model 1	Model 2	Model 3	Model 4
		GMR (95%CI); * p* value	GMR (95%CI); * p* value	GMR (95%CI); * p* value	GMR (95%CI); * p* value
LMR	Low-SES	Reference	Reference	Reference	Reference
	High-SES	0.78 (0.68–0.90); * p* < 0.001	0.76 (0.65–0.88); * p* < 0.001	0.818 (0.671–1.000); *p* = 0.049	0.73 (0.61–0.88); * p* = 0.001
I-FABP	Low-SES	reference	reference		
	High-SES	0.81 (0.7–0.94); * p* = 0.004	0.83 (0.71–0.97); * p* = 0.016		
LBP	Low-SES	Reference	Reference		Reference
	High-SES	1.22 (1.01–1.47); * p* = 0.036	1.30 (1.07–1.60); * p* = 0.010		1.40 (1.12–1.73); * p* = 0.003

Multivariate analysis using linear regression models. Model 1: crude. Model 2: adjusted for age, sex, z-BMI; Model 3: Model 2 + *A. lumbricoides* infection. Model 4: Model 2 + *D. fragilis* infection. SES: socioeconomic status. GMR: Geometric Mean Ratio. CI: Confidence Interval. LMR: Lactulose Mannitol Ratio; I-FABP: Intestinal Fatty Acid Binding Protein; LBP: LPS Binding Protein.

Furthermore, we observed no association between z-BMI and any of the gut biomarkers (Supplementary Table S3). In addition, the effect of SES on all these markers did not change after adjusting for z-BMI (Table 2).

### Effect of albendazole treatment on parasitic infections and gut microbiota

In low-SES group, albendazole treatment resulted in a reduction of proportion of subjects infected with helminths and in infection intensity. Albendazole had the largest effect on *A. lumbricoides*, followed by *T. trichiura* (Fig. 1a). Before treatment, the percentage of moderate and light infection intensity for *A. lumbricoides* were 10.1% and 36.7% while 3.8% and 29.1% for *T. trichiura*, respectively. Following treatment, only light infections were seen for these two parasites and the proportion of those infected with *A. lumbricoides* and *T. trichiura* was reduced to 3.8% and 8.9%, respectively. In high-SES group, we found no helminth-infected children following albendazole administration. Treatment also led to a reduction in the prevalence of *G. lamblia* (from 51.7% to 31.7%, p.adj = 0.005 in low-SES; from 23.3% to 10.6%, p.adj = 0.04 in high-SES) but changes were not observed for other protozoa (Fig. 1b).

**Figure 3:**
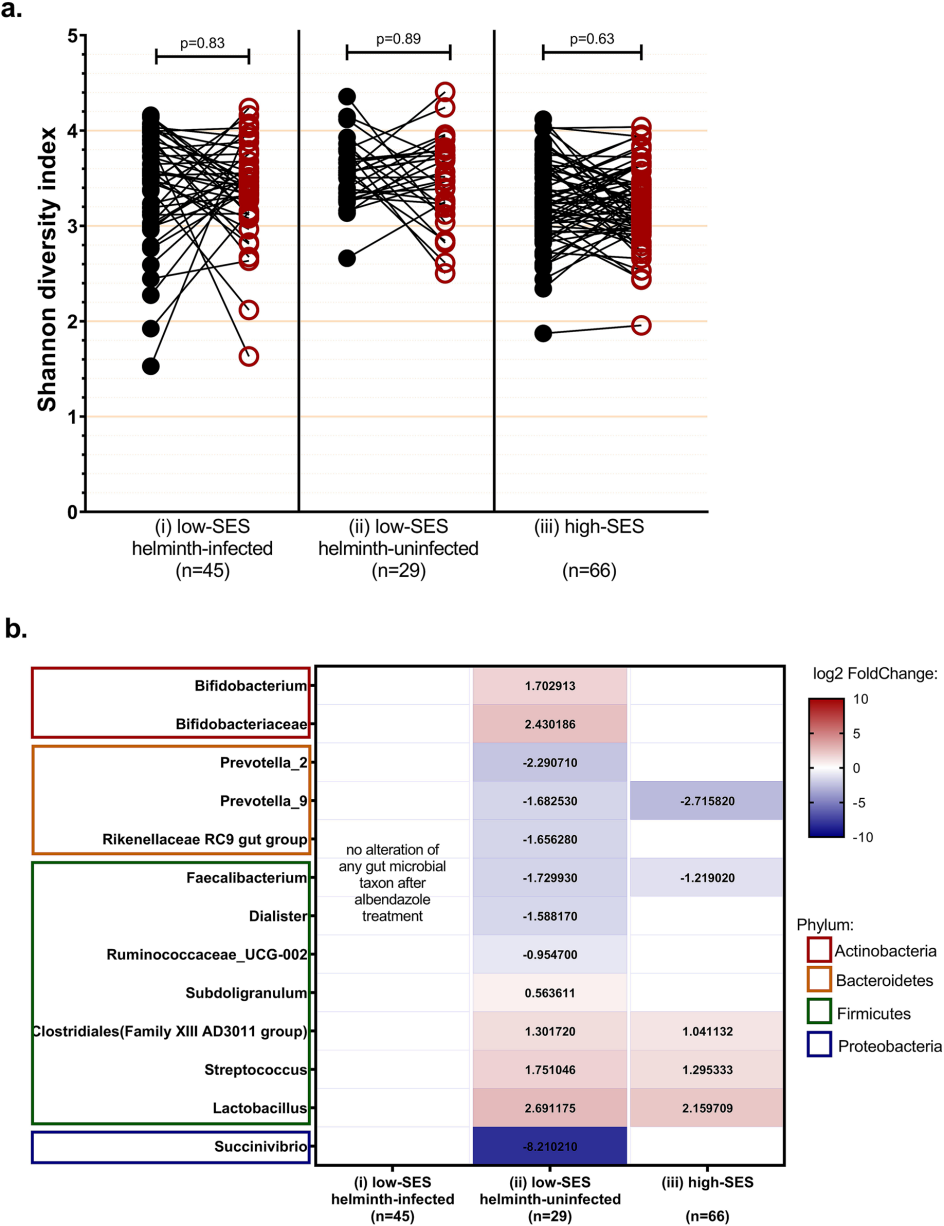
Effect of triple-dose albendazole treatment on (**a**) gut microbiota diversity; and b) gut microbiota composition in (i) low-SES helminth-infected, (ii) low-SE helminth-uninfected, and (iii) high-SES children. (**a**) Shannon diversity index measurements at both timepoints were compared using Wilcoxon signed rank test. Black closed-dots: before treatment; red open-dots: after treatment. (**b**) Data plotted as log_2_ fold change derived from differential abundance analysis by DESeq2. Cell colours indicate taxa changes after albendazole treatment: red colour indicate increased relative abundance after treatment and blue colour indicate decreased relative abundance after treatment. Only taxa detected to have significant difference in abundance (adjusted *p* value  < 0.05) are displayed; adjusted *p* value were determined using Benjamini–Hochberg method. Row annotation showed specific taxa that were assigned under 4 different phylum. SES = Socioeconomic status.

The differences in the diversity and composition of bacterial gut microbiota at baseline between high and low-SES children of this study have been published^13^. These children shared a core microbiota consisting of *Bifidobacterium*, *Collinsella*, and multiple members of *Lachnospiraceae* and *Ruminicoccaceae* families, but the diversity and the relative abundance of several taxa differed depending on SES^13^. At baseline, Shannon diversity index was higher in the low- compared to the high-SES; however, no differences were seen when we compared helminth-infected vs -uninfected in low-SES^13^. In line with this, after treatment, there were no changes in Shannon diversity index in low-SES children, whether these were helminth-infected or not, nor in high-SES children group (Fig. 3a). Similarly, we observed no alteration in gut microbiota composition in helminth-infected low-SES children after treatment. However, we observed some alteration of several short chain fatty acid (SCFA)-producing bacteria in the uninfected low- and high-SES children. We found decreased *Faecalibacterium* and *Prevotella*, but an increased *Lactobacillus, Streptococcus, and Clostridiales* relative abundance in the low-SES uninfected and high-SES children after albendazole treatment (Fig. 3b). In addition, some other bacteria changed in the same group: a decreased relative abundance of *Succinivibrio*, *Dialister,* and *Rikenellaceae,* and increased relative abundance of *Bifidobacteriaceae* and *Bifidobacterium* in low-SES helminth-uninfected children (Fig. 3b).

### Effect of albendazole treatment on intestinal barrier function

**Figure 4:**
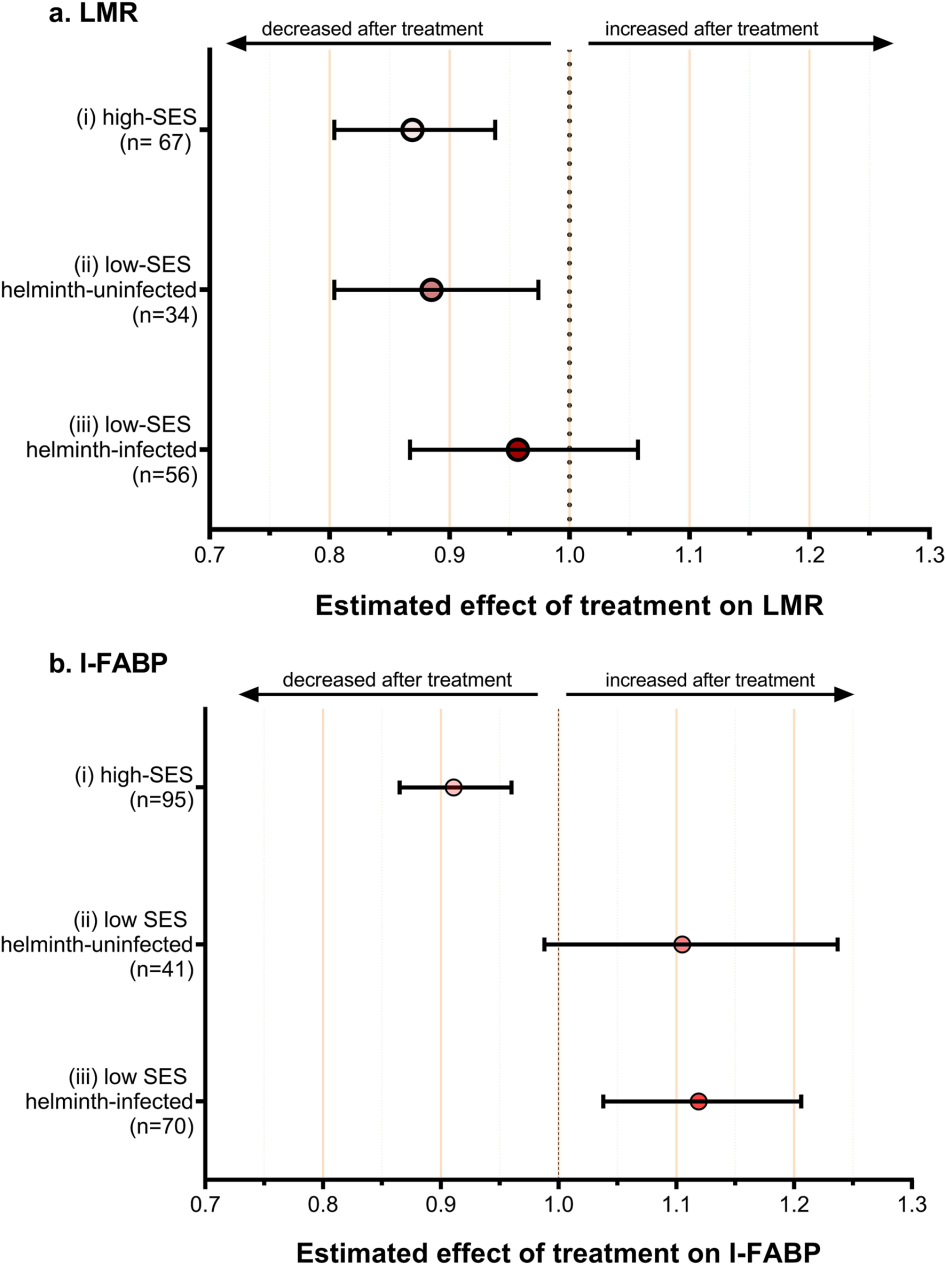
Effect of albendazole treatment on (**a**) LMR and (**b**) I-FABP in study children stratified by SES and helminth infection at baseline (i) high-SES (ii) low-SES helminth-uninfected; and (iii) low-SES helminth-infected. Analysis was using linear mixed model and adjusted for age, sex, and zBMI. The estimated treatment effects are presented as geometric means ratios with the corresponding 95% confidence interval.

As shown in Fig. 4a, albendazole significantly decreased LMR in helminth-uninfected children. Estimated treatment effect was largest in high-SES children, with a 13% reduction (p.adj < 0.001), while in low-SES uninfected children there was a 11% reduction (p.adj = 0.01). No significant reduction was observed in low-SES children who were helminth-infected at baseline.

Similar analysis was performed for I-FABP, marker of intestinal damage (Fig. 4b). Firstly, we observed a 9% decrease in I-FABP in high-SES children (p.adj < 0.001). In contrast, there was an increase of 12% (p.adj = 0.004) in I-FABP following albendazole treatment in low-SES helminth-infected children and although a similar increase was seen in the I-FABP in uninfected low-SES children, this fell short of statistical significance (p.adj = 0.08).

Regarding the protozoa, when the model was adjusted for these infections, the effect of albendazole on LMR and I-FABP did not change. Albendazole treatment did not alter the levels of LBP.

To answer the question whether the effect of treatment on LMR and I-FABP was mediated by a specific gut microbiota, we analysed the correlation between the relative abundance of specific taxa and these biomarkers. We could not pinpoint one specific bacterial taxon that might contribute to markers of gut barrier function nor to intestinal damage. (Supplementary Figure S3).

## Discussion

In this study, we demonstrated that intestinal barrier function, measured by LMR, I-FABP, and LBP differs between low- and high-SES schoolchildren living in an urban center of Makassar, Indonesia. Low-SES children exhibited higher LMR and I-FABP, yet lower LBP. The higher LMR, indicating increased intestinal permeability and higher I-FABP, a marker of epithelial damage, show that the intestinal barrier function and integrity might be compromised in low- compared to the high-SES children. We had hypothesized that high intestinal permeability might allow bacterial translocation and result in higher LBP levels. Contrary to this, we observed higher LBP values in high SES children that were not associated with zBMI, which could mean that LBP is a better marker of inflammation associated with other factors, such as macronutrient intake. A longitudinal study of healthy lean subjects has shown an increase in LPS and LBP concentrations in subjects given a high-fat, high-carbohydrate meal but not in subjects given a high-fiber and fruit meal^14^.

In answer to our question whether the differences were due to intestinal parasitic helminths, which were different between low- and high-SES children, we assessed the effect of reducing helminth infections through albendazole treatment. The differences in LMR or I-FABP could not be attributed to current intestinal helminth infections. While LMR decreased after treatment with albendazole, this was restricted to children who were uninfected at baseline. In contrast, I-FABP, increased after albendazole treatment, which might indicate that killing and expulsion of worms, leads to more epithelial damage. In addition, although we observed some changes in microbiota composition after the administration of albendazole, the differences in gut integrity markers could not be explained by differences in microbiota composition. These data indicate that although there are significant differences in gut barrier function of low and high SES children, this could not be accounted for by current parasitic infections.

Despite deworming programs in Indonesia, helminth infections are still widespread, especially in low-SES children. In addition, several parasitic protozoa, such as *G. lamblia*, are more common in low-SES compared to high-SES children in our urban Indonesian population, in line with other studies^15–17^. Several studies have explored the effect of helminth infections on intestinal permeability, however only one has considered SES as a contributing factor^11^. *A. lumbricoides* infection was associated with elevated LMR in Bangladeshi and Malaysian children^11,12^, consistent with our findings in the crude analysis. However, multivariate analysis including both SES and *A. lumbricoides* indicated that SES is the main driver of the difference in LMR. Other factors than current *A. lumbricoides* infections could contribute to increased LMR, for example recurrent gastrointestinal infections that lead to diarrhea^18^. Intestinal permeability can be assessed by measuring several sugar probes such as lactulose, sucrose and sucralose^5^. Sucrose is thought to reflect gastroduodenal permeability, meanwhile sucralose indicates colonic permeability^5,19^. In this study, lactulose mannitol ratio was used to describe the gut function of each participant which reflects paracellular passage in the small intestines where most of the soil-transmitted helminths reside. An important other advantage of LMR over the other markers is that the urine collection is less time-consuming making it, therefore more suitable for use in children.

It should be noted that exercise can also induce intestinal damage or intestinal permeability, especially after a long and intense physical activity such as running or cycling for 90 min^20^. Several studies have shown that active school transport such as walking have been associated with higher physical activity in general^21^, which might contribute to the higher LMR in low-SES children.

I-FABP, expressed in mature intestinal epithelial cells, is released into circulation if the cell membranes are damaged^22,23^. I-FABP has been used as a non-invasive marker for acute intestinal damage or integrity loss^24^. In our study, we found no influence of *Ascaris* and *Trichuris* infections on I-FABP. This is in contrast to studies in subjects infected with hookworm and *Strongyloides stercoralis,* which reported elevated levels of I-FABP^25,26^. It is possible that the latter two helminths are more pathogenic, for example, hookworms feed on intestinal mucosa and blood^27^, which can indeed result in more damage. In our study, the marked increased I-FABP after treatment in helminth-infected children, might suggest that removal of worms leads to local inflammation and damage to epithelial cells, however, as there was a tendency for a similar effect in the helminth-uninfected low-SES children, it is possible that albendazole either directly or indirectly affects gut epithelial homeostasis and damage.

One of the biomarkers generated in response to bacterial translocation is LBP, a class 1 acute phase protein. Unexpectedly, in our study, LBP was found to be lower in low-SES where the intestinal barrier was more disrupted compared to high-SES children. The higher LBP levels in high-SES schoolchildren might be in line with previous observations where elevated LBP levels were linked to obesity, weight gain^28,29^, high fat and high carbohydrate diet^14^, as in the high-SES, the children have a different nutritional status and some are obese. However, these differences persisted after controlling for zBMI, suggesting there may be other factors at play, potentially high-fat and high-carbohydrate intake^14,30^ Another factors to consider is that LPS translocation is not necessarily only through paracellular leakage but also through transcellular transport^31^, however, the clinical relevance of this transcellular pathway is still unclear.

As published already, gut microbiota composition of Indonesian schoolchildren in our study is associated with socioeconomic status, even when living in the same urban area^13^. Not only sanitation^32^ but also differences in diet, hygiene, or helminth infections might be causes of the differences in the gut microbiota profile^32–35^. Regarding the diversity of microbiota in our population, we have reported that low-SES children have higher microbial diversity compared to high-SES children, independently of helminth-infection status^13^. Easton and colleagues showed that 3 weeks after albendazole treatment gut microbiota diversity did not change, yet, microbiota composition was altered with decreased *Aeromonodales (Gammaproteobacteria)*. However, their study did not distinguish the effect of albendazole, between helminth infected and uninfected subjects^36^. In line with finding of Easton and colleagues, we showed no change in microbiota diversity while microbiota composition was altered 4 weeks after albendazole administration. Nonetheless, larger sample size in our study allowed us to stratify the population based on their SES and helminth status, where the relative abundance of several taxa was altered but only in the uninfected subjects and not in helminth-infected subjects of low-SES group. Moreover, we also observed changes in the high-SES children, indicating that alteration in bacterial taxa was more SES-related and not associated with helminth infections. Further studies should shed light on whether the altered microbiota composition, in a SES-specific manner, is albendazole-related or reflects natural oscillations over time.

An important limitation of our study is the substantial loss to follow-up. Despite our efforts to retain children within the study, 48% of children could not be followed up. However, we report no difference in baseline characteristics between the children that were lost to follow up and those who remained in the study. Furthermore, although we controlled for pre-identified confounders, dietary intake and physical activity was not surveyed, thus, their effect on intestinal permeability and microbiota composition could not be explored. No placebo was used in our study, consequently, we do not have the benefits of controlling for the changes related to time rather than treatment. Due to the lack of data regarding urine volume in this study, we are not able to compare the LMR result with published data and it is not possible to report the level of lactulose or mannitol urinary excretion.

In conclusion, the level of intestinal permeability and acute intestinal injury as measured by LMR, as well as LBP and I-FABP, differed between high- and low-SES children, and these differences were not associated with intestinal parasitic infections. Further research is needed to elucidate the exact mechanisms responsible for the elevated intestinal permeability in low-SES children as well as the off-targets effect of albendazole.

## Material and methods

### Study population and design

The study was conducted in Makassar, South Sulawesi, Indonesia. Ethical approval was obtained through the local ethics committee of Hasanuddin University (approval number: 1504/H04.8.4.5.31/PP36-KOMETIK/2016). This study was conducted in accordance with the Declaration of Helsinki. Study participants were recruited from two elementary schools that are distinct in SES. The low-SES school was located near the port area where mostly low-skilled labourers lived and worked. Children generally lived in the area surrounding the school site and travelled to school on foot. The high-SES school was located in the city centre. Children attending this school lived in distinct parts of the city, mostly in residential compounds and travelled to school by privately chartered school buses or by private vehicles.

The primary outcome of this study was intestinal permeability, as measured using LMR, while the secondary outcome was the intestinal damage and bacterial translocation, as assessed using I-FABP and LBP; after albendazole treatment in high and low SES schoolchildren. Based on previously reported data, we calculated that around 84 children were required in each group to detect a 25% relative difference in LMR (mean difference of 0.1, SD = 0.02) with a significance level of 5% (2-tailed) and a power of 90%.

Prior to the start of the study, an information letter concerning the study was given to the parents seeking permission for their children to participate in the study. Only children who returned the signed informed consent were included in the study. At baseline, anthropometry data were collected. Blood sample was obtained from median cubital vein by a venipuncturist. The day before, a stool container with enclosed spoon (Sarstedt AG&Co.KG, Nümbrecht, Germany) was given to these children. They were asked to collect stool samples in the morning before school, the same day when the study was conducted.

As soon as samples were gathered by research staff on site, stool, blood and urine samples were stored inside an ice box and transported to the Laboratory of Parasitology Department at Hasanuddin University and Hasanuddin University Medical Research Center laboratory to be aliquoted and kept at − 80˚C for further analyses.

After completion of the baseline visit, all participants received a single albendazole dose (400 mg, PT HoliPharma, Cimahi, Indonesia) given for three consecutive days regardless of their helminth infection status. The follow-up visit took place 4 weeks after treatment at which collection of blood, stool, and urine samples were repeated.

### Parasitological examination

A single Kato-Katz slide was prepared from each stool sample^37^ and examined for the detection of STH Infection. Intensity was determined for each species according to WHO guidelines^38^. PCR was performed to identify intestinal protozoa. Briefly, DNA was extracted from stool samples followed by a multiplex real-time PCR used for the specific amplification and detection of *Entamoeba histolytica*, *Dientamoeba fragilis*, *Giardia lamblia*, and *Cryptosporidium parvum*. The procedure has been described elsewhere^39–41^. PCR output was expressed as the cycle threshold (Ct)-value reflecting the load of specific DNA in the sample tested. Protozoa specific DNA load were categorized into low load (35 ≤ Ct < 50), moderate load (30 ≤ Ct < 35), or high load (Ct < 30). Negative DNA results were recoded as Ct = 50.

### Urinary lactulose-mannitol ratio (LMR)

Following overnight fasting, a lactulose/mannitol drink, containing 2 g mannitol and 5 g lactulose dissolved in 100 mL drinking water, was administered at school. The following three hours, all urine was collected in a large container together with 1 mL chlorhexidine 2% as a preservative. Urine samples were analysed using liquid chromatography mass spectrometry (LC–MS) as described previously^42,43^. LMR was calculated by dividing the lactulose concentration by the mannitol concentration in absence of data on the collected urine volumes. These values were multiplied with 100 to create percentages.

### ELISA for measurements of I-FABP and LBP

ELISA techniques were used to quantify the concentrations of I-FABP and LBP (Duoset, R&D system, UK), according to the manufacturer’s instruction. For these assays, serum was diluted 8 and 2000 times for I-FABP and LBP, respectively. The results were expressed in ng/ml for I-FABP and µg/ml for LBP.

### Microbiota analysis

Microbiota analysis was performed in 140 children from whom sufficient stool samples were available before and after treatment. The procedure for sample processing and microbiota analysis is already described elsewhere^13^. Raw sequencing data are available in the European Nucleotide Archive (https://www.ebi.ac.uk/ena) under study accession PRJEB38465 (baseline) and PRJEB40889 (follow up).

### Statistical analysis

In accordance with the WHO guidelines, age standardized of z-scores of body mass index (z-BMI) were calculated. For the crude analyses, categorical data were compared using chi-square tests, whereas normally distributed continuous data was compared using the student t-test. Correlation between variables was tested using Pearson or Spearman correlation and we considered ρ ≥ 0.4 suggestive for a relevant correlation. To help explore the complex interplay between SES and helminth infection, the analysis was stratified into i) high-SES, ii) low-SES helminth-uninfected, and iii) low-SES helminth-infected. Linear regression models were used to adjust for a priori confounders such as age, sex, and z-BMI in addition to the identified explanatory variables. The data was analysed using IBM SPSS Statistics version 25 (IBM‐SPSS Inc., Chicago, USA), and GraphPad Prism (GraphPad Software, Inc., CA, US) was used for visualisation. Longitudinal data were analysed using mixed models with subject random effects, and fitted using lmerTest package (R software)^44^. LMR, I-FABP, and LBP were log10-transformed before analysis. Parameter estimates of treatment effects (95%CI) were then back transformed to obtain the geometric mean ratios (GMR). The reported *p* values were obtained using a likelihood ratio test comparing the model with and without a time effect.

Microbiota data were analysed in R-software (v3.5.1) using the packages phyloseq (v1.26.1)^45^, vegan (v2.5–4)^46^, ggplot2 (v3.1.0)^47^, DESeq2 (v1.22.2)^48^ and microbiome (v1.4.2)^49^. Wilcoxon signed rank tests were performed to compare Shannon diversity index before and after treatment groups. For differential abundance testing by DESeq2, the OTU-table was filtered for OTUs present in less than 25% of the samples to minimize zero-variance errors and spurious significance. Outcomes were considered significant when the Benjamini–Hochberg corrected *p* value was ≤ 0.05. To analyse albendazole-altered taxa in this population, paired analysis was done.

### Conference presentation

Keystone Symposia “Helminths: New Insights from Immunity to Global Health”, Cape Town, South Africa, December 2019. Abstract number: 1003 (A.I.A.).

## Supplementary Information

Supplementary Information.
