# Supplementary material for: Intestinal permeability before and after albendazole treatment in low and high socioeconomic status schoolchildren in Makassar, Indonesia
Source: Sci Rep. 2022 Mar 1;12:3394. doi: 10.1038/s41598-022-07086-7 (PMC8888571; doi:10.1038/s41598-022-07086-7)
Supplement: Supplementary file 1 — Supplementary Information. [file 41598_2022_7086_MOESM1_ESM.pdf]

# SUPPORTING INFORMATION

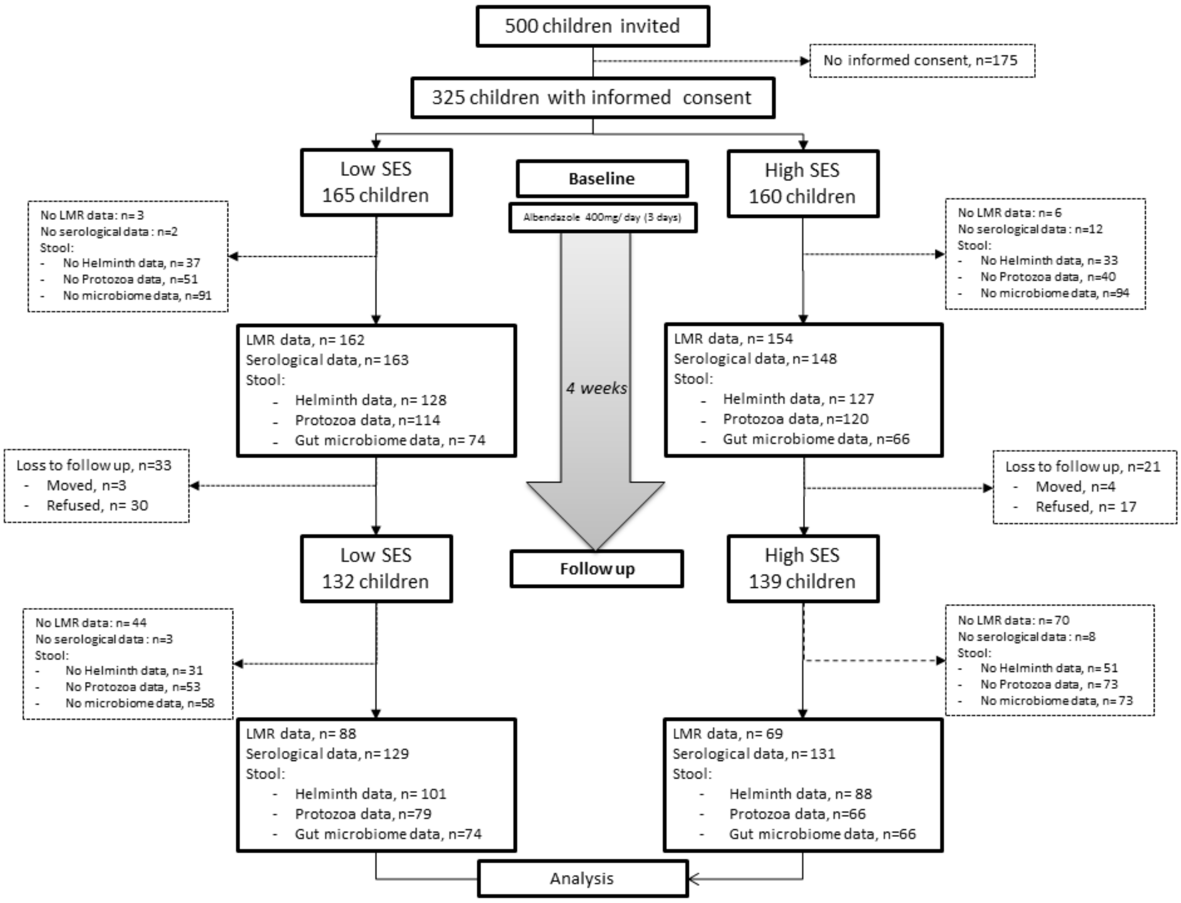


**Supplementary Figure S1.** Consort Diagram. After collecting baseline data all participant (n=325) were treated with single dose albendazole for three consecutive days. Follow up data were collected 4 weeks after albendazole treatment. SES: socioeconomic status. LMR = lactulose mannitol ratio.

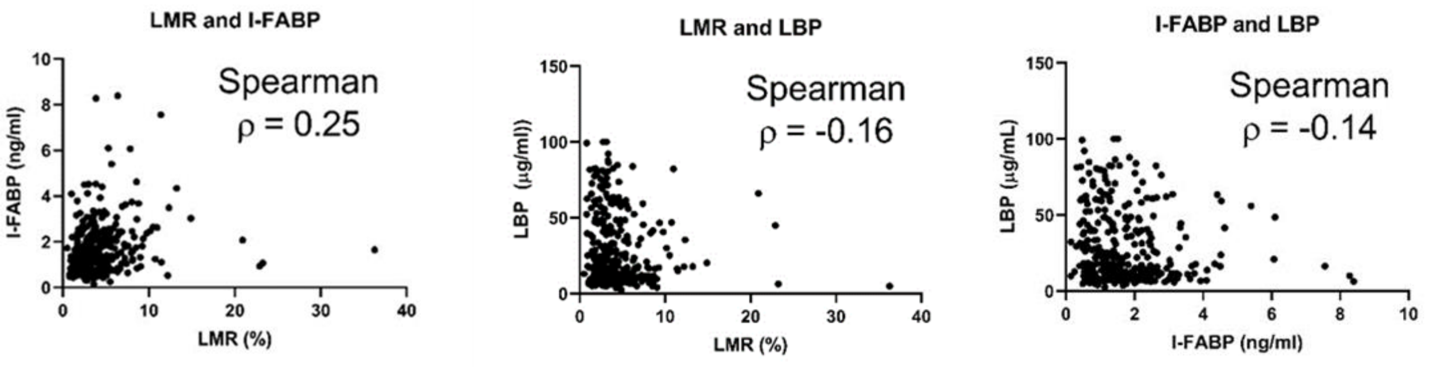


**Supplementary Figure S2.** Correlation between paired biomarkers at baseline. Data presented as ρ (spearman correlation) and considered relevant correlation if ρ≥0.4. LMR: Lactulose Mannitol Ratio; I-FABP: Intestinal Fatty Acid Binding Protein; LBP: LPS Binding Protein.

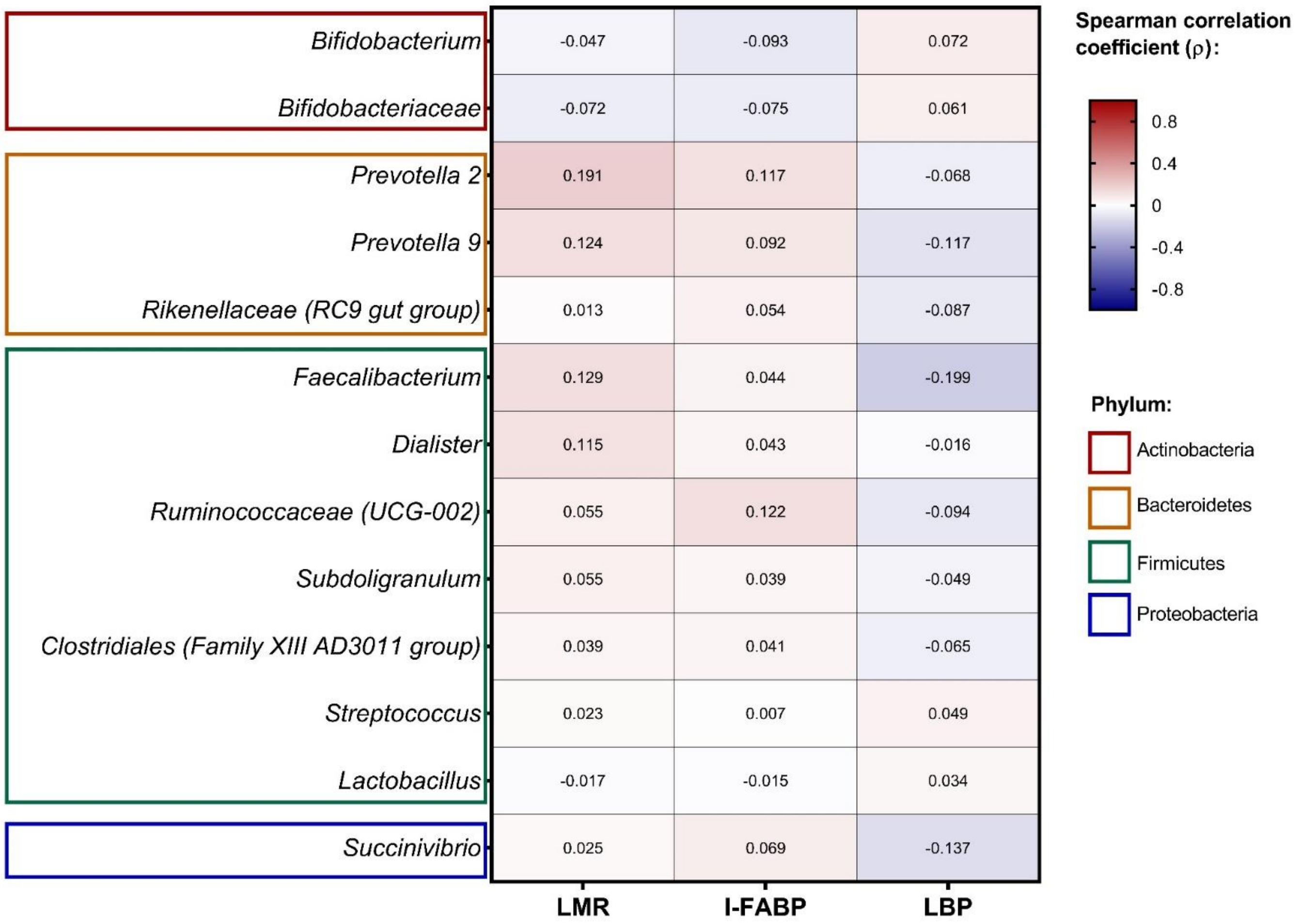


**Supplementary Figure S3.** Correlation between albendazole-altered taxa and LMR, I-FABP, and LBP. Data presented as ρ (spearman correlation) and considered relevant correlation if ρ≥0.4. LMR: Lactulose Mannitol Ratio; I-FABP: Intestinal Fatty Acid Binding Protein; LBP: LPS Binding Protein.

**Supplementary Table S1.**

Characteristics of study population remained in the study and loss to follow-up

| Characteristics | remained in the study | lost to follow up | p-value |
|---|---|---|---|
| **SES (low-SES), n/N, %** | 88/157 (56.1) | 74/159 (46.5) | 0.093 |
| **Any helminths, n/N, %** | 58/157 (36.9) | 27/90 (30.0) | 0.330 |
| **Sex (female), n/N, %** | 93/157 (59.2) | 81/159 (50.9) | 0.143 |
| **Age, mean ± SD (N)** | 10.30 ± 0.87 (157) | 10.23 ± 0.90 (159) | 0.501 |
| **zBMI, mean ± SD (N)** | -0.36 ± 1.47 (157) | -0.31 ± 1.47 (159) | 0.755 |

The number of positives (n) of the total population examined (N). SD: standard deviation. Statistical testing was performed using student t-test for continuous variables and using chi-square test for categorical variables.

**Supplementary Table S2.** Geometric means and 95% confidence intervals for gut permeability markers in relation to different explanatory variables

| | LMR (%) | | I-FABP (ng/ mL) | | LBP (µg/mL) | |
|---|---|---|---|---|---|---|
| | N | Geomean (95%CI) | | Geomean (95%CI) | N | Geomean (95%CI) |
| **Any intestinal helminth** | | | | | | |
| **Positive** | 85 | 4.15 (3.60 - 4.78) | 85 | 1.55 (1.33 – 1.79) | 85 | 15.91 (13.46 – 18.81) |
| **Negative** | 162 | 3.56 (3.24 – 3.93 | 158 | 1.35 (1.22 – 1.49) | 159 | 18.80 (16.64 – 21.23) |
| ***Ascaris lumbricoides*** | | | | | | |
| **Positive** | 58 | **4.44 (3.74 – 5.28)** | 59 | 1.51 (1.25 – 1.83) | 59 | 17.10 (13.86 – 21.10) |
| **Negative** | 189 | **3.57 (3.26 – 3.90)** | 184 | 1.38 (1.26 – 1.52) | 185 | 17.94 (16.04 – 20.07) |
| ***Trichuris trichiura*** | | | | | | |
| **Positive** | 53 | 4.16 (3.40 – 5.08) | 53 | 1.54 (1.27 – 1.88) | 53 | 16.55 (13.27 – 20.64) |
| **Negative** | 194 | 3.65 (3.35 – 3.98) | 190 | 1.38 (1.26 – 1.51) | 191 | 18.08 (16.19 – 20.19) |
| **Any intestinal protozoa** | | | | | | |
| **Positive** | 127 | 3.83 (3.42 - 4.30) | 126 | 1.38 (1.22 – 1.56) | 125 | 19.03 (16.63 – 21.78) |
| **Negative** | 101 | 3.60 (3.18 – 4.06) | 97 | 1.46 (1.28 – 1.65) | 99 | 15.89 (13.60 – 18.54) |
| ***Entamoeba histolytica*** | | | | | | |
| **Positive** | 18 | 4.07 (3.09 – 5.38) | 18 | 1.43 (0.99 – 2.08) | 18 | 15.60 (10.60 – 19.74) |
| **Negative** | 210 | 3.70 (3.39 – 4.04) | 205 | 1.41 (1.29 – 1.55) | 206 | 17.75 (15.97 – 19.74) |
| ***Dientamoeba fragilis*** | | | | | | |
| **Positive** | 64 | 3.64 (3.05 – 4.36) | 64 | 1.31 (1.10 -1.56) | 63 | **20.84 (17.19 – 25.28)** |
| **Negative** | 164 | 3.76 (3.42 – 4.13) | 159 | 1.45 (1.31 – 1.66) | 161 | **16.44 (14.58 – 18.51)** |
| ***Giardia lamblia*** | | | | | | |
| **Positive** | 144 | 3.86 (3.35 – 4.46) | 140 | 1.44 (1.23 – 1.70) | 142 | 19.50 (16.51 – 23.05) |
| **Negative** | 84 | 3.65 (3.29 – 4.04) | 83 | 1.40 (1.26 – 1.55) | 82 | 16.54 (15.55 – 18.81) |

CI: confidence intervals. Testing performed using unpaired student t-test on log transformed data. Bold: p-value<0.05. LMR: Lactulose Mannitol Ratio; I-FABP: Intestinal Fatty Acid Binding Protein; LBP: LPS Binding Protein

**Supplementary Table S3.** Association between anthropometric measurement and LMR, I-FABP, and LBP.

| | | LMR | I-FABP | LBP |
|---|---|---|---|---|
| zBMI | GMR (95%CI) | 1.02 (0.97-1.07) | 0.97 (0.92-1.03) | 0.95 (0.88-1.01) |
| | p.adj | 0.497 | 0.371 | 0.119 |
| zHA | GMR (95%CI) | 1.04 (0.97-1.11) | 0.96 (0.90-1.03) | 0.95 (0.86-1.04) |
| | p.adj | 0.320 | 0.304 | 0.231 |

Data presented as geometric mean ratio (GMR) with 95% confidential interval (95%CI). Adjusted p-value (p.adj) was derived from linear regression analysis and adjusted for SES, age and sex. LMR: Lactulose Mannitol Ratio; I-FABP: Intestinal Fatty Acid Binding Protein; LBP: LPS Binding Protein.
